# Complete uterine inversion during caesarean section: A case report

**DOI:** 10.1186/1757-1626-1-127

**Published:** 2008-08-27

**Authors:** Dimitrios Vavilis, Dimitrios Tsolakidis, Dimitrios Athanatos, Antonios Goutzioulis, John N Bontis

**Affiliations:** 1 First Department of Obstetrics and Gynaecology, Aristotle University of Thessaloniki, 'Papageorgiou' Hospital, Thessaloniki, Greece

## Abstract

Inversion of the uterus through the uterine lower segment incision during a caesarean section is an extremely rare obstetric incident. It consists, though, an emergency complication that is potentially life-threatening, especially in cases of prolonged inversion, because haemodynamic instability and shock may occur. Prompt diagnosis and immediate uterine reversion are the key actions in the management of this serious complication.

## Introduction

Inversion of the uterus through the uterine incision during cesarean section is a rare, but potentially life-threatening emergency. When repositioning of the uterus is not immediate, excessive bleeding can cause haemodynamic instability and shock, that need proper resuscitation. To the best of our knowledge no more than 13 reports of this intraoperative complication have been found in the literature [1-13].

We present a case of uterine inversion during caesarean section, that was managed successfully. It is noted that this is the first case in our department reported the last 15 years.

## Case presentation

A 29-year-old healthy gravida two, para one with previous caesarean section underwent a lower segment caesarean section at 36 weeks for premature labor under general anaesthesia.

Patient history details were as follows: Occupation: housewife; Ethnicity: Greek; Weight: 87 Kgr; Height: 167 cm; Medical history: one previous caesarean section, otherwise unremarkable; Family history: unremarkable; Patient habits and medication: non-smoker, no alcohol consuption, vitamin supplement during pregnancy.

**Figure 1:**
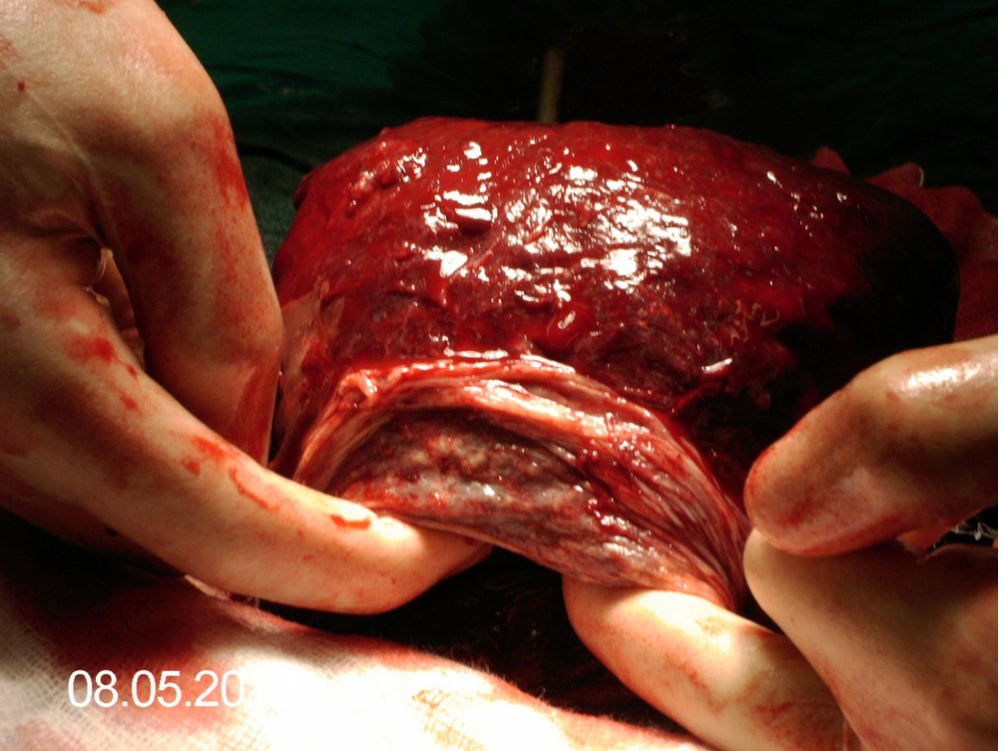
Complete uterine inversion with fundally implanted placenta (Top: fundus of the uterus, bottom: cervical contraction ring).

Delivery of the fetus was uneventful. After the baby was born, an intravenous bolus of 10 i.u of oxytocin was administered. Uterine contraction was noted and gentle cord traction was applied in order to remove the placenta. With slight cord traction, complete inversion of the uterus, through the uterine incision occurred with the placenta remaining firmly attached to the uterine fundus (Fig. 1). The inverted uterus was exteriorized at once and the placenta was manually removed. Several attempts for uterine reversion were done unsuccessfully for less than five minutes. Eventually, sevoflurane anesthesia was deepened from 1% to 5% and reversion of the uterus was finally achieved by gradually rolling the lowermost part of the posterior edge over the uterine fundus, thereby reverting that part that inverted last. The uterus was repositioned intraabdominally and an infusion of 20 i.u oxytocin plus 0.2 mg methylergometrine in 1000 ml Ringer's Lactated set maintained the uterine contractions. Sevoflurane progressively was reduced to the initial concentration. Uterine closure was followed by closure of the abdominal cavity. No significant changes in the haemodynamic status of the patient were noted during the operation. Blood loss was estimated at 1500 ml and two units of whole blood were transfused. After the end of cesarean section, 0.8 mg of misoprostol was given per rectum. The postoperative period was uneventful and the patient was discharged from the hospital on the 4^th ^postoperative day.

## Discussion

The exact incidence of uterine inversion during caesarean delivery in not known, but it seems to be an extremely rare complication. It is reported that the incidence is much lower than that of uterine inversion following vaginal delivery and occurs in approximately one out of 1860 caesarean sections [1], but we believe that this is overestimated because it is the first case reported in our department the last years. Remarkably noted that from the few cases reported in the literature there are two unique incidents of cervical inversion [13] and uterine torsion of an inverted uterus [11] during caesarean section.

The causes of this complication remain unclear. Fundal insertion of the placenta [2], inherent weakness of the uterine musculature [2,3], the administration of oxytocin, in particular when given as a bolus [3,4] and traction of the cord with the placenta, either partially or completely attached to the uterus (adherent placenta) [4-6], could be probable contributing factors of this complication.

Uterine inversion is a serious and potentially life-threatening complication. The principal features of this complication are haemorrhage and shock. The blood loss depends on the inversion-reversion interval and can lead to serious haemodynamic instability [3,7,8]. It has been supported that hypotension and shock may be neurogenic in origin, owing to the traction on the patient's infundibulopelvic ligaments or secondary to peritoneal or broad ligament stretching [4]. However, given that the patient is under anaesthesia, either general or regional, the neurogenic element of shock should be considered as eliminated [2], so the blood loss remains the main reason for the patients' instability. Management of uterine inversion during caesarean section is usually simple, if diagnosed promptly within a few minutes. In case of a delay in diagnosis and uterus reversion, it may lead to hypotension and difficulty in repositioning the uterus, resulting possibly in fatal outcome [2,3].

The administration of volatile anaesthetic agent in high concentrations, such as halothane or sevoflurane, may facilitate the rapid repositioning of the uterus producing uterine relaxation. The exact effect of those two agents is dose-dependent depression of contractility and developed tension of human myometrium [9,14].

In our case controlled cord traction was followed immediately by complete inversion. The administration of bolus oxytocin, the firm fundal insertion of the placenta, and the cord traction, although controlled and gentle could have been contributing factors. Of course, an inherent uterine musculature weakness cannot be excluded, but this is extremely difficult to be proven. Anaesthesia deepening by increasing sevoflurane concentration facilitated the manual uterine reposition. Prompt diagnosis of the complication and rapid manipulation of the uterus facilitated an successful outcome.

In conclusion, uterine inversion during cesarean section is a serious and often unexpected obstetric complication. The obstetrician should be aware of this complication. Prompt diagnosis and uterine reversion without any delay are the key in the management of this life-threatening obstetric emergency.

## Consent

Written informed consent was obtained from both patients – in their native language – for publication of this case report and accompanying image. Copies of the written consent are available for review by the Editor-in-Chief of this journal.

## Competing interests

The authors declare that they have no competing interests.

## Authors' contributions

DV conceived the study and participated in patient management, acquisition of data, interpretation of data, and was a major contributor in writing the manuscript. DT participated in patient management, acquisition of data, and drafting of the manuscript. DA revised critically the manuscript adding substantial intellectual content. AG participated in patient management, acquisition of data, and drafting of the manuscript. JB coordinated the study and patient management and revised critically the manuscript. All authors have read and approved the final manuscript. The manuscript is not under consideration and has not been published by another journal.
